# The Endocrine Society Issues Statement of Principles

**DOI:** 10.1289/ehp.120-a346

**Published:** 2012-08-31

**Authors:** Charles W. Schmidt

**Affiliations:** Charles W. Schmidt, MS, an award-winning science writer from Portland, ME, has written for *Discover Magazine*, *Science*, and *Nature Medicine*.

A new position statement from The Endocrine Society provides a strong argument for scientists in industry, government, and academia to work together, across disciplines, to improve testing of chemicals as potential endocrine disruptors. Published ahead of print in *Endocrinology* on 25 June 2012,^1^ the statement focuses on the Environmental Protection Agency’s (EPA) Endocrine Disruptor Screening Program (EDSP) to illustrate how fundamental endocrinology principles might be incorporated into more rigorous screens for endocrine activity. But according to lead author R. Thomas Zoeller, a biology professor at the University of Massachusetts, Amherst, the need for broader consideration of endocrinology extends to screening programs beyond the EDSP.

The EDSP flags potential endocrine disruptors with a two-tiered system. Tier 1 assays, which were validated by the Federal Insecticide, Fungicide, and Rodenticide Act Science Advisory Panel in 2008,^2^ provide information for use in identifying chemicals that could potentially interact with the endocrine system. Tier 2 assays, which are currently undergoing validation, evaluate dose responses between those interactions and chemical exposure. The Endocrine Society statement focuses on Tier 1, which employs a variety of *in vitro* and *in vivo* assays with a long history in toxicology.

Given that they mimic hormones, endocrine disruptors don’t behave like other toxicants, Zoeller explains. Chemical effects generally increase with greater exposure, but hormones rarely display dose linearity. That’s because if a hormone saturates its receptor, more of the same hormone will have no greater effect on response. And in some cases, high doses downregulate responses triggered by lesser exposures—in other words, different effects can appear and disappear at different doses. These “nonmonotonic” dose–response curves—commonly accepted in endocrinology—challenge a fundamental premise of toxicology: that chemical effects become more pronounced with increasing dose.

The Endocrine Society’s view is that exposure levels employed in the EDSP’s Tier 1 assays—which start with the maximum tolerated doses identified in previous toxicology testing, then work their way down—are so high as to potentially miss low-dose, nonmonotonic effects. But Lorenz Rhomberg, principal at Gradient Corporation, an environmental risk assessment firm in Cambridge, Massachusetts, counters that Tier 1 tests were designed solely to test for endocrine system interactions, so the dose doesn’t matter. “Questions about dose response are dealt with in Tier 2,” he says. In response to e-mailed questions regarding the EDSP, an EPA spokesman^3^ writes that the Agency’s Office of Research and Development is “developing a review of the state of the science on low-dose, nonmonotonic dose–response curves and its potential impact on . . . risk assessment.”

The definition of the term “endocrine disruption” is an important factor in determining the evidence needed to identify a chemical as an endocrine disruptor. The World Health Organization (WHO) and the European Union define endocrine disruptors specifically as causing adverse effects in organisms, whereas the EPA and the WHO cite specific mechanisms by which endocrine disruption occurs. The Endocrine Society, on the other hand, proposes a streamlined version of the EPA’s 1996 definition of an endocrine disruptor to include “exogenous chemicals or chemical mixtures that interfere with any aspect of hormone action,” irrespective of adverse effects or specific mechanisms.^1^

**Figure:**
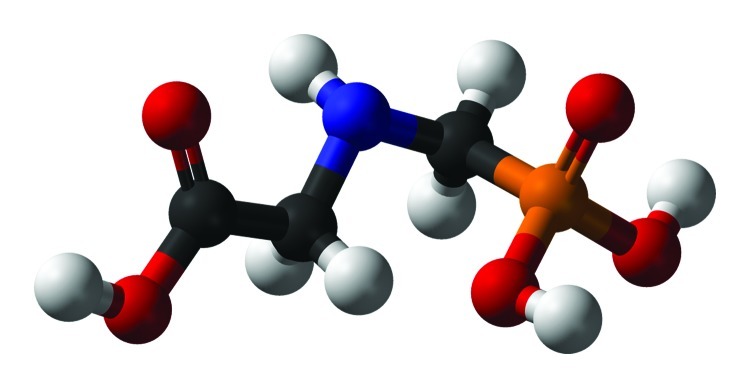
Given that they mimic hormones, endocrine disruptors don’t behave like other toxicants. Hormones rarely display dose linearity, and in some cases, high doses downregulate responses triggered by lesser exposures—in other words, different effects can appear and disappear at different doses. Image courtesy of U.S. EPA

The statement explains that screening only for a limited set of end points that does not reflect full understanding of endocrinology principles means bona fide endocrine disruptors could slip through undetected. For instance, by judging thyroid hormone action primarily on the presence of histopathological changes in the thyroid—which typically are a function of changes in serum levels of thyroid-stimulating hormone (TSH)—the EDSP does not recognize other important means by which the thyroid influences health. Polychlorinated biphenyls reduce levels of thyroxine but not of TSH. If these chemicals were to undergo Tier 1 testing today, they likely would not be flagged as endocrine-disrupting chemicals for study in Tier 2.^1^

George Gray, director of the Center for Risk Science and Public Health at the George Washington University, in Washington, DC, says the proposed definition is toxicologically unorthodox. “It’s going to need a lot of scientific vetting,” he says. “This is hardly something that should be adopted by EPA without a lot of consultation with scientists both within and outside the agency.” The EPA spokesman said the agency would follow peer-review risk assessment methodology and evaluate chemicals on a case-by-case basis in its efforts to set regulatory points of departure that protect human health.

A total of 67 chemicals have been selected for Tier 1 screening so far, at a cost of $500,000 each, according to the agency spokesman. The agency continues to review incoming data, but none of these chemicals—most of them pesticide active ingredients and high-production-volume chemicals used as inert ingredients in pesticide formulations—have been flagged as endocrine disruptors using current tests.
